# Liquid‐Suspendable Metabolic Sensors Allow Real‐Time Oxygen Consumption Monitoring in Caco‐2 and Colon Organoid‐Based Gut‐on‐a‐Chip Models

**DOI:** 10.1002/biot.70333

**Published:** 2026-11-04

**Authors:** Sarah Devriese, Flavio Bonanini, Anna F. Bekebrede, Sara Shafiei, Dorota Kurek, Coen Govers, Hans Bouwmeester, A. Paul Vos, Remko van Vught, Jaap Keijer, Vincent C. J. de Boer

**Affiliations:** ^1^ Human and Animal Physiology Wageningen University Wageningen The Netherlands; ^2^ Mimetas B.V. Oegstgeest The Netherlands; ^3^ Cell Biology and Immunology Wageningen University Wageningen The Netherlands; ^4^ Division of Toxicology Wageningen University Wageningen The Netherlands; ^5^ Department of Food, Health and Consumer Research Wageningen Food & Biobased Research Wageningen The Netherlands

**Keywords:** Caco‐2, colon organoids, metabolic sensor, organ‐on‐a‐chip, oxygen consumption rate, time‐resolved fluorescence

## Abstract

The organ‐on‐a‐chip field is a rapidly evolving area of expertise, combining enhanced physiological relevance with a wide range of functional readouts across diverse cellular models. One such readout is oxygen metabolism, which serves as a key indicator of mitochondrial function. In this study, we utilized metabolic sensors designed for real‐time measurement of oxygen consumption, reflecting aerobic respiration, within a commercially available microfluidic platform. We demonstrated that time‐resolved detection of oxygen consumption is feasible within the system's microliter‐scale volumes and that chemically induced changes in cellular oxygen metabolism can be monitored in real time in two ready‐to‐use gut‐on‐a‐chip models. In summary, this novel application of metabolic sensors enables direct evaluation of mitochondrial function in complex cellular environments by addressing key analytical challenges often encountered in organ‐on‐a‐chip systems due to their limited size and structural complexity.

## Graphical Abstract and Lay Summary

A soluble oxygen sensor measures real‐time cell respiration inside a closed gut‐on‐a‐chip with a standard plate reader, without modifying the chip. In Caco‐2 and colon organoid models, this gives a full mitochondrial stress test and makes metabolic measurements possible in commercial organ‐on‐a‐chip devices.

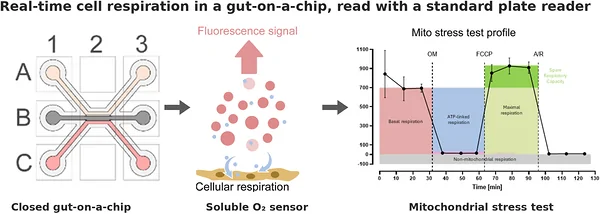

**Abbreviations**
5PL: 5 parameter logistic
A/R: antimycin A/rotenone
ECM: extracellular matrix
EMEM: eagle's minimum essential medium
FBS: fetal bovine serum
FCCP: carbonyl cyanide p‐trifluoro‐methoxyphenyl hydrazone
GOX: glucose oxidase
HBSS: Hank's balanced salt solution
NEAA: non‐essential amino acids
OCR: oxygen consumption rate
OM: oligomycin
OoC: organ‐on‐a‐chip
OW: observation window
RFU: relative fluorescence unit
SD: standard deviation
SRC: spare respiratory capacity
TEER: transepithelial electrical resistance
TRF: time‐resolved fluorescence

## 1 Introduction

Organs‐on‐a‐chip (OoC) are miniaturized complex cell systems grown inside microfluidic devices [1]. These devices use spatial modeling and flow‐based shear stress to emulate cell‐ and tissue‐specific microenvironments and functions. Due to this high level of physiological mimicry, OoCs are emerging as the next‐generation experimental platforms to investigate human (patho‐) physiology, to evaluate the effects of pharmacological and nutritional compounds with increased clinical predictivity, and to replace animal testing [2, 3]. In addition to structural tissue modeling, it is therefore crucial that OoC systems can also recapitulate physiologically relevant parameters in vitro.

One of these parameters is mitochondrial metabolic activity. Mitochondria provide the cell with chemical energy, stored as ATP, as well as metabolic and redox components, and are a signaling hub for regulating cellular fate and functionality decisions [4]. Optimal mitochondrial function is essential for physiological health and its dysfunction is a hallmark of aging and a wide range of chronic diseases such as intestinal diseases, cardiovascular diseases, cancer and neurodegeneration [5, 6, 7, 8, 9, 10]. In inflammatory bowel diseases specifically, emerging evidence suggests that cross‐talk between gut microbiota and host mucosal mitochondria alters mitochondrial metabolism, activates immune cells, induces inflammasome signaling and promotes loss of epithelial barrier integrity [11].

Oxygen consumption is the key proxy for mitochondrial functionality and activity, since most of the cellular oxygen is used as the final electron acceptor at the end of the mitochondrial electron transport chain in mammalian cells [12]. Measuring oxygen consumption is thus crucial for understanding cellular metabolism and physiological functionality, which is relevant for disease modeling, drug discovery and toxicity testing. Oxygen consumption in 2D well plate models is commonly measured using analytical platforms where the oxygen consumption rate (OCR) is detected via plate‐embedded sensors [13]. In contrast, measuring oxygen in 3D OoC systems requires either custom‐made chip systems that contain such embedded sensors, and are thus specifically designed for this purpose, or fiber‐optic microsensor probes that can be inserted into the microfluidic device, which is not always feasible due to chip design specifics [14, 15, 16, 17, 18, 19, 20, 21, 22]. An overview of these oxygen measurement methods and their main limitations for organ‐on‐a‐chip use is given in Table 1.

**TABLE 1:** Oxygen and metabolic monitoring approaches for cell and organ‐on‐a‐chip systems. For each method the table lists the measuring principle, whether the sensor must be built into the chip, the spatial detail, the invasiveness, and the main limitation for use in organ‐on‐a‐chip systems. The soluble sensor used in this study requires no sensor built into the chip and no probe inserted into the channel.

Method	Principle	Chip integration	Spatial resolution	Invasiveness	Real‐time	Multiparameter	Main limitation for OoC	Reference
Extracellular flux (Seahorse XF)	Transient microchamber, solid‐state O_2_/pH flurophores	None; dedicated well plates	None, per well	Static microchamber, not microfluidic	Yes	O_2_+pH	Not designed for microfluidic or 3D tubular OoC; needs dedicated instrument	[13, 27]
Integrated O_2_ sensor spots (optodes)	Luminescent spots fixed in the chip, read optically	Built in at fabrication	Point, per spot	Non‐invasive once integrated	Yes	O_2_	Requires custom chip design	[19, 41]
Planar sensor foils	O_2_‐sensitive foil at the channel floor, imaged from below	Built in at fabrication	2D map over foil	Non‐invasive once integrated	Yes	O_2_	Requires foil integrated and compatible imaging	[16]
Fiber‐optic microprobe	Needle‐type optode inserted into the channel	Access port needed	Point, at probe tip	Invasive; can perturb flow	Yes	O_2_	Insertion not feasible in many closed chip	[17, 21]
Integrated O_2_/pH sensors	On‐chip multiparameter sensors	Built in at fabrication	Point or defined region	Non‐invasive once integrated	Yes	O_2_+pH	Requires microfabrication and added read‐out	[18, 20]
Soluble suspendable sensor (this work)	Cell‐impermeable phosphorescent probe dissolved in the medium	None; added to the medium	Per chamber	Non‐destructive; flow interruption needed	Yes	O_2_ (pH variant exists)	Small‐volume signal; static measurement	[23, 24, 25, 29]

Here we propose the use of a commercially available oxygen‐sensing reagent as a non‐invasive way of measuring oxygen consumption, which can be used irrespective of the type of OoC device. The metabolic sensor used in this study is chemically stable, inert, cell impermeable and liquid‐suspendable, allowing suspension into the cell culture medium [23, 24, 25]. The sensor's phosphorescent signal is quenched by oxygen through molecular collision and is therefore inversely proportional to the amount of extracellular oxygen present. The reaction between oxygen and sensor is non‐destructive and transient, thus enabling continuous measurements. We first demonstrated the feasibility of detecting the sensor's signal from the small solution volume inside the microfluidic channels of a commercially available gut‐on‐a‐chip model [26]. We then developed an OCR determination workflow for a biological context by using the OoC system with human intestinal epithelial Caco‐2 tubules or adult stem cell‐derived colon organoid tubules grown inside, coupled with the sequential addition of established mitochondrial respiration modulators [13, 27].

## 2 Materials and Methods

### 2.1 Cell Culture

The human colorectal adenocarcinoma cell line Caco‐2 (LGC Standards; cat. no. ATCC‐HTB‐37) was grown in Eagle's Minimum Essential Medium (EMEM; LGC Standards; cat. no. ATCC‐30‐2003) supplemented with 10% heat‐inactivated fetal bovine serum (FBS; Thermo Fisher Scientific; cat. no. 10500064), 1% non‐essential amino acids (NEAA; Thermo Fisher Scientific; cat. no. 11140050) and 1% of a penicillin‐streptomycin solution containing 10,000 units/ml of penicillin and 10,000 µg/ml of streptomycin (Thermo Fisher Scientific; cat. no. 15140122), hereafter referred to as complete EMEM. Cells were passaged at around 80% confluency using 0.25% trypsin‐EDTA (Thermo Fisher Scientific; cat. no. 25200072) and subcultured at a concentration between 5 × 10^3^ and 10 × 10^3^ cells/cm^2^. Cells were maintained at 37°C in a humidified atmosphere of air/CO_2_ (95:5, v/v) with 1 to 2 medium changes per week.

### 2.2 Gut‐on‐a‐Chip Culture

On‐chip culture was performed using the OrganoPlate 3‐lane 40 (Mimetas; cat. no. 4004‐400‐B) according to the manufacturer's protocol [26]. To start, all wells in the observation window lanes were filled with 50 µl of Hank's Balanced Salt Solution (HBSS; Thermo Fisher Scientific; cat. no. 14025050) to prevent dehydration and to provide optical clarity when imaging the observation window (Figure 1Aii, well “B2”). Next, around 2 µl of extracellular matrix (ECM) was loaded into the gel inlets (Figure 1Aii, well “B1”) using a Picus electronic pipette (Sartorius; cat. no. 735021). The ECM was composed of an 8:1:1 mixture of 5 mg/ml collagen I (Cultrex 3‐D Culture Matrix Rat Collagen I; Bio‐Techne; cat. no. 3447‐020‐01), 1 M HEPES (Thermo Fisher Scientific; cat. no. 15630049) and 37 g/l NaHCO_3_ dissolved in sterile MilliQ water with a pH adjusted to 9.5 using NaOH (Sigma‐Aldrich; cat. no. S5761), yielding a final concentration of 4 mg/ml collagen I, 100 mM HEPES and 3.7 g/l NaHCO_3_. After incubation for 15 min in a humidified incubator (37°C; 5% CO_2_), 20 µl of HBSS was added on top of the gel inlets and the plate was incubated overnight in a humidified incubator (37°C; 5% CO_2_). The following day, Caco‐2 cells were trypsinized, pelleted (300 g; 5 min; room temperature) and counted. A Caco‐2 cell suspension was prepared in complete EMEM at a concentration of 1 × 10^4^ cells/µl. Subsequently, Caco‐2 cells were seeded into the microfluidic device using a process called wet seeding. Briefly, HBSS was removed from all gel inlets and 50 µl of complete EMEM was added to every top medium outlet (Figure 1Aii, well “A3”). Next, 2 µl of the Caco‐2 cell suspension was injected into every top medium inlet (Figure 1Aii, well “A1”). The observation window was used to check for proper cell seeding and adequate cell numbers. The plate was subsequently incubated on its side for 3–4 h in a humidified incubator (37°C; 5% CO_2_) to allow the cells to sediment and attach to the ECM. Afterwards, 50 µl of complete EMEM was added to all top medium inlets as well as to all bottom medium inlets and outlets (Figure 1Aii, wells “C1” and “C3”). The plate was then placed horizontally in a humidified incubator (37°C; 5% CO_2_) on an interval rocker switching between a +7° and –7° inclination every 8 min (OrganoFlowL; Mimetas; cat. no. MI‐OFPR‐L), allowing bidirectional flow. With these settings, a mean flow rate of 2.02 µl/min could be achieved, resulting in a mean shear stress of 0.13 dyne/cm^2^. This result has previously been considered representative of physiological intestinal shear stresses, which range from 0.002 to 0.08 dyne/cm^2^ [28]. The medium was refreshed the day after seeding and then every other day. Commercially available, ready‐to‐use gut‐on‐a‐chip models with either pre‐seeded Caco‐2 tubules (OrganoReady Colon Caco‐2 3‐lane 64; Mimetas; cat. no. MI‐OR‐CC‐02) or pre‐seeded adult stem cell‐derived colon organoid tubules (OrganoReady Colon Organoid 3‐lane 64; Mimetas; cat. no. MI‐OR‐CORG‐02) were cultured according to manufacturer's instructions.

**FIGURE 1:**
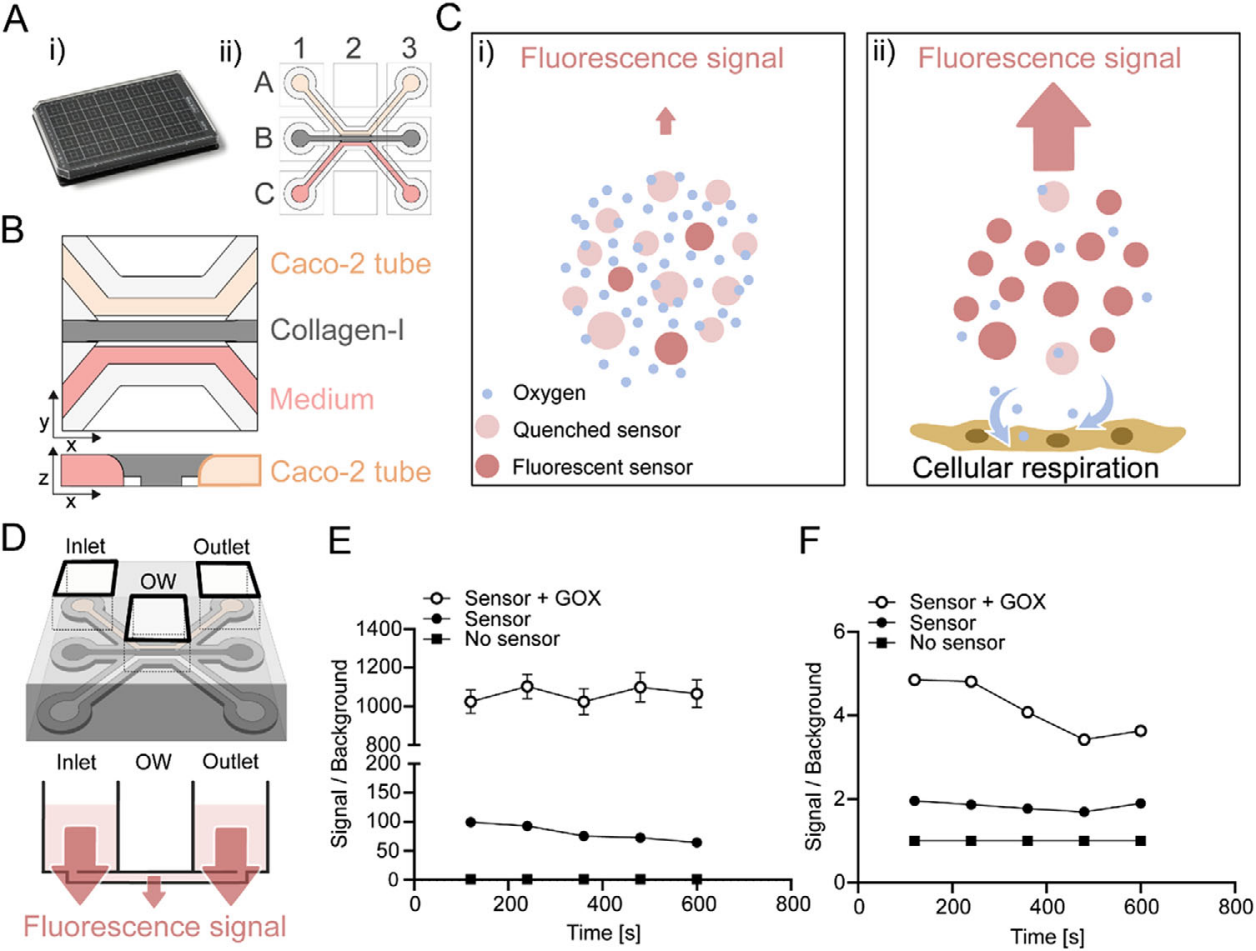
Metabolic sensor detection in an empty microfluidic device. (A) The OrganoPlate 3‐lane 40 (i) patterned with 40 individual chip units with microfluidic channels connecting the different wells (ii). (B) Schematic of the observation window (OW) and a typical culture set‐up; a cellular tubule in the top channel adjacent to an ECM‐filled middle channel. (C) Working principle of the MitoXpress Xtra metabolic sensor. In an oxygen‐rich solution, oxygen molecules quench the sensor's fluorescence (i), whereas in presence of oxygen‐consuming cells, the fluorescent signal increases over time (ii). (D) Schematic of the connections between inlets, outlets and OW within one chip unit. Large arrows indicate the high fluorescence signal originating from the inlets and outlets, whereas a lower signal emanates from the OW. (E) Raw fluorescence intensity measured from the medium inlets and outlets of an empty chip unit containing 50 µl of complete EMEM each, either without (Background) or with (Signal) sensor and glucose oxidase (GOX). Mean ± SD of N = 4. (F) Raw fluorescence intensity measured from the OW of an empty chip unit, either without (Background) or with (Signal) sensor and GOX. N = 1.

### 2.3 Transepithelial Electrical Resistance Measurements

Barrier integrity of the Caco‐2 tubules was determined by measuring transepithelial electrical resistance (TEER) using an automated multichannel impedance spectrometer (OrganoTEER; Mimetas) according to the manufacturer's instructions. OrganoTEER determines TEER from the impedance spectrum and reports the values directly as area‐normalized resistance (Ω·cm^2^), using the ECM‐supported cell area for normalization. For the collagen I ECM used in this study, the estimated cell‐covered area is approximately 0.0057 cm^2^, as specified for the OrganoTEER system. As specified by the manufacturer, TEER is extracted from the raw impedance spectra without requiring baseline correction. Cell‐free negative control chips containing ECM but no cells were included to assess background electrical resistance.

### 2.4 Time‐Resolved Extracellular Oxygen Consumption Measurements

Oxygen consumption inside the microfluidic device was analyzed using the MitoXpress Xtra Oxygen Consumption Assay (Agilent Technologies; cat. no. MX‐200‐4) according to the manufacturer's instructions. Fluorescence intensity measurements, quantified as relative fluorescence units (RFU), were performed using the Tecan Spark multimode microplate reader in the time‐resolved fluorescence (TRF) detection mode. Fluorescence measurements for all cellular experiments were performed only within the observation window (Figure 1Aii, well “B2”). Parameter settings are listed in Table 2. Changes in oxygen consumption were quantified from changes in the sensor's fluorescence signal over time and expressed as RFU/min, providing a fluorescence‐derived relative measure of oxygen consumption, hereafter referred to as oxygen consumption rate (OCR). Caco‐2 mitochondrial respiration modulators (Seahorse XF Mito Stress test; Agilent Technologies; cat. no. 103015–100) were used at final concentrations of 1.5 µM for oligomycin (OM), 1 µM for carbonyl cyanide p‐trifluoro‐methoxyphenyl hydrazone (FCCP) and 0.5 µM for antimycin A/rotenone (A/R). Glucose oxidase (GOX; Sigma‐Aldrich; cat. no. 345386) was used at a final concentration of 2 units/ml. All experiments were conducted in complete EMEM. After each measurement cycle, the plate was positioned at a 7° angle in a humidified incubator (37°C; 5% CO_2_) for 1 min to allow re‐oxygenated medium to perfuse through the microfluidic channels. The plate was then kept static for an additional minute prior to the start of the next measurement cycle.

**TABLE 2:** Settings used for the time‐resolved fluorescence intensity measurement parameters.

Parameter	Setting
Temperature	37°C
Optics position	Bottom
Filters (wavelength/bandwidth)	Excitation: 350 nm/35 nm Emission: 665 nm/8 nm
Integration time	30 µs
Lag time	70 µs
Settle time	100 ms
Gain	144
Z‐position	30565 µm
Number of flashes	10

### 2.5 Statistical Analysis

Statistical analysis and illustration of results were performed using GraphPad Prism version 10.0.1 for Windows (GraphPad Software, Boston, Massachusetts USA). Data normality was determined using the Kolmogorov–Smirnoff test. Differences between groups were analyzed using an unpaired t‐test in case of normally distributed data (applying the Welch's correction in case of unequal variances) or the Mann–Whitney statistic if the data were not normally distributed. A p‐value of <0.05 was considered statistically significant. Data are presented as mean ± standard deviation (SD).

A 5 parameter logistic (5PL) regression was used to fit the fluorescence data obtained over time (*t*) for the measurement cycles at baseline or in presence of FCCP using the following equation:

$$f\;\left(t\right)=Bottom+\frac{Top-Bottom}{{\left[1+\left(2^{\frac{1}{S}}-1\right)\cdot {\left(\frac{C}{t}\right)}^{Hillslope}\right]}^{S}}$$

Where: Bottom = Minimum asymptote, Top = Maximum asymptote, Hillslope = Hill's slope, C = Inflection point, S = Asymmetry factor.

The obtained parameters were then used to calculate the maximum OCR, described as the derivative with respect to time (t) by using the following equation:

$$\frac{d}{dt}f\;\left(t\right)=\frac{\left(Top-Bottom\right)\cdot S\cdot \left(2^{\frac{1}{S}}-1\right)\cdot Hillslope\cdot {\left(\frac{C}{t}\right)}^{Hillslope}\cdot \frac{1}{t}}{{\left[1+\left(2^{\frac{1}{S}}-1\right)\cdot {\left(\frac{C}{t}\right)}^{Hillslope}\right]}^{S+1}}$$

Wells in which oxygen saturation was not reached, which is required for the calculation of the ‘Top’ parameter, were excluded from the maximum OCR calculation.

For OM and A/R measurement cycles, the raw data did not follow a typical Hill equation. Therefore, a linear regression was used to fit the data obtained in presence of OM or A/R using the following equation:

$$f\;\left(t\right)=a\;\cdot t+b$$

And its derivative with respect to time (*t*):

$$\frac{d}{dt}f\;\left(t\right)=a$$

Where *a* = Slope and *b* = Intersect.

## 3 Results

### 3.1 Metabolic Sensor Detection via Time‐Resolved Fluorescence in an Organ‐on‐a‐Chip System

We initially evaluated the feasibility of obtaining a detectable fluorescence intensity signal from the metabolic sensor (Figure 1C) within the organ‐on‐a‐chip (OoC) system (Figure 1A) by conducting measurements from both the inlets and outlets (Figure 1D), as well as from the observation window (Figure 1B,D) of an empty chip unit. To this end, 50 µl of complete EMEM with or without the metabolic sensor was added to all medium inlets and outlets (Figure 1Aii; wells “A1”, “A3”, “C1” and “C3”). In addition, we included glucose oxidase (GOX) as a positive control to maximize the sensor's signal, since GOX depletes oxygen by metabolizing the glucose within the cell culture medium. We found that the sensor's fluorescence intensity from the inlets and outlets increased a 100‐fold compared to the background signal from those without the sensor, which was further increased to approximately a 1000‐fold with the addition of GOX (Figure 1E). Both signal‐to‐background ratios exceeded the manufacturer's requirement for the sensor, which specifies that the signal should be at least 3‐fold higher than the background. The microfluidic channels connected to all inlets and outlets converge beneath the observation window (Figure 1Aii; well “B2”), where the accumulated volume is around 3 microliters. Here, the increase in signal‐to‐background fluorescence upon addition of the sensor was approximately twofold or fourfold without or with GOX, respectively (Figure 1F).

### 3.2 Mitochondrial Respiration Modulation in an Organ‐on‐a‐Chip System With Caco‐2 Cells

Next, we assessed whether oxygen consumption could be detected within chip units that contained cells. To this end, Caco‐2 cells were cultured for 9 days in the top channel of the OoC system (Figure 2A). Leak tightness of the intestinal epithelial barrier was confirmed, as indicated by an average transepithelial electrical resistance (TEER) of 750 Ω.cm [2] (Figure 2B). To measure oxygen consumption, we developed a workflow comprising the acquisition of fluorescence measurements every 10 s over a 10‐minute period under static (no‐flow) conditions. Each measurement was followed by a brief manual tilt of the plate in order to re‐oxygenate the medium (Figure 2C). This measurement cycle was repeated for a total of 3 times to establish a baseline. Next, different modulators—inhibitors or activators—of cellular respiration; oligomycin (OM), carbonyl cyanide p‐trifluoro‐methoxyphenyl hydrazone (FCCP) and antimycin A/rotenone (A/R), were tested sequentially. For every modulator, 3 measurement cycles were performed, each separated by re‐oxygenation through plate tilting. After completing 3 cycles with one modulator, the medium in all inlets and outlets was replaced before introducing the next modulator. This enabled the determination of mitochondrial function parameters (basal, maximal, ATP‐linked and non‐mitochondrial respiration, proton leak and spare respiratory capacity) by selectively inhibiting/activating various components of the electron transport chain. Specifically, OM inhibits ATP synthase, A/R inhibit complexes I and III, respectively and FCCP is a potent mitochondrial uncoupler (Figure 2D). Under basal conditions, we observed a marked increase in fluorescence over time, which plateaued after approximately 7 to 8 min for each of the three measurement cycles, likely indicating a gradual depletion of oxygen in the medium through Caco‐2 respiration (Figure 3A). Plate tilting performed after each measurement cycle caused a drastic signal reduction, indicating that new, re‐oxygenated medium had been introduced into the channels. The addition of OM significantly reduced the signal increase over time, an effect that was reversed by subsequent FCCP treatment and ultimately abolished again following A/R addition. We observed that for basal and FCCP conditions, the signal followed a sigmoidal curve which could be fitted with a 5PL curve, whereas OM and A/R conditions could be fitted using a linear model (Figure 3B). As we ultimately aimed at measuring the rate of oxygen consumption, we calculated the first derivative of the fitted curves relative to time, demonstrating the changes in maximal OCR after consecutive use of the different mitochondrial modulators (Figure 3C). Plotting the maximal OCR of each measurement cycle yielded a typical Mito Stress test profile, from which basal, maximal, ATP‐linked and non‐mitochondrial respiration, proton leak and spare respiratory capacity could be inferred (Figure 3D).

**FIGURE 2:**
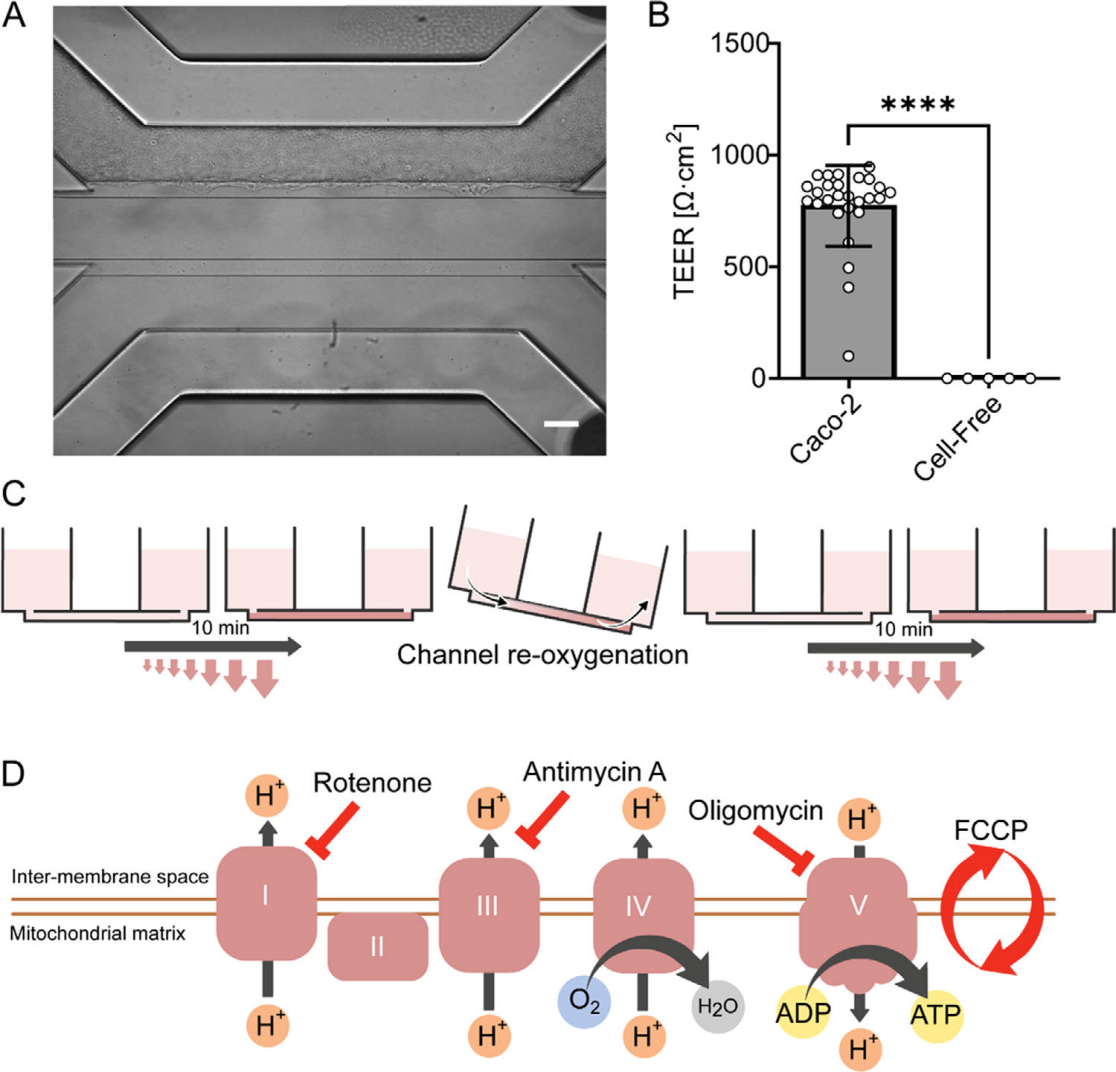
Caco‐2 mitochondrial respiration modulation: assay set‐up. (A) Phase‐contrast image of a Caco‐2 tubule grown in the top channel after 9 days of culture. Scalebar = 200 µm. (B) Transepithelial electrical resistance (TEER) measurement at day 9 of culture prior to the assay. Mean ± SD of N = 27 (Caco‐2) or N = 5 (Cell‐Free). ****p < 0.0001. (C) Workflow illustration. Each cycle consists of fluorescence measurements acquired every 10 s over a 10‐min period under static (no‐flow) conditions. This leads to a gradual oxygen depletion within the microfluidic channel, as illustrated by the transition from pink to red shading and the increasing downward motion of the red arrows. Each measurement period is followed by tilting the plate for 1 min on a rocker. Angular tilting of the plate creates a gravity‐driven medium perfusion as shown by the directionality of the arrows. This allows re‐oxygenation of the medium inside the microfluidic channel as illustrated by the gradual disappearance of the red shading. The plate is then held static for an additional minute before the next measurement cycle begins to allow the medium within the inlets and outlets to equilibrate. (D) Illustration of the mitochondrial electron transport chain with the different modulator targets. Rotenone and Antimycin A inhibit complexes I and III, respectively to shut down mitochondrial respiration and measure non‐mitochondrial baseline respiration. Oligomycin inhibits complex V (ATP synthase) to measure ATP‐linked respiration. FCCP uncouples oxidative phosphorylation by collapsing the proton gradient to drive maximal respiration.

**FIGURE 3:**
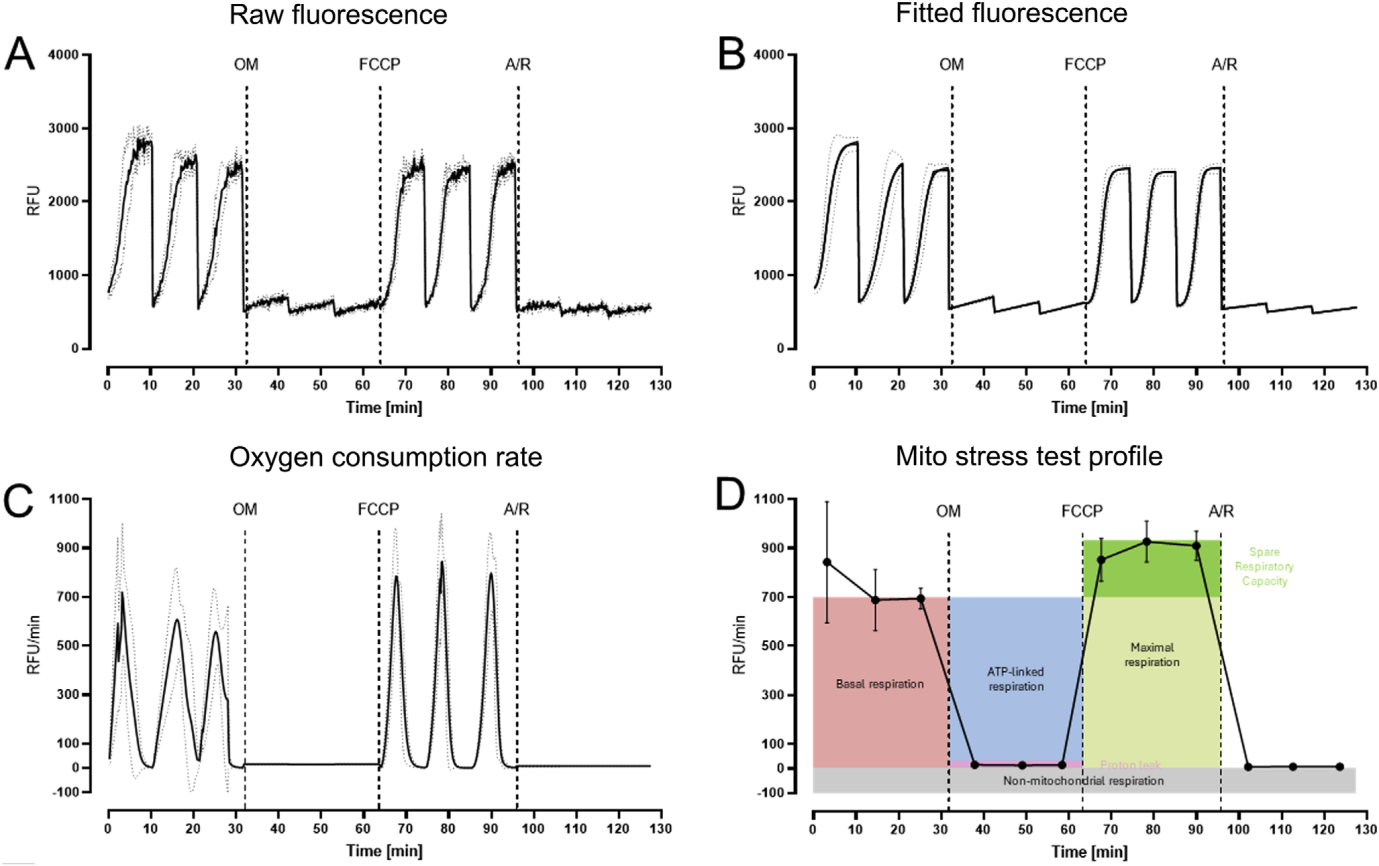
Caco‐2 mitochondrial respiration modulation: Mito Stress test. (A) Raw fluorescence intensity expressed as relative fluorescence units (RFU) over time across 3 measurement cycles per modulator (OM; oligomycin, FCCP; carbonyl cyanide p‐trifluoro‐methoxyphenyl hydrazone, A/R; antimycin A/rotenone). Mean (full lines) ± SD (dotted lines) of N = 3, with N = number of chip units. (B) Fitted models on the raw data. A 5PL fit was used for basal and FCCP cycles, a linear fit was used for OM and A/R cycles. (C) First derivative with respect to time, showing oxygen consumption rates (OCR). (D) Mito Stress test profile obtained by plotting the maximal OCR of each measurement cycle. Mean (dots) ± SD (error bars) of N = 3, with N = number of chip units. Timepoints of compound addition are indicated by dashed lines.

### 3.3 Mitochondrial Respiration Modulation in Ready‐to‐Use Gut‐on‐a‐Chip Models

Finally, we assessed whether a similar OCR pattern using the same respiration modulators could also be observed in two commercially available, ready‐to‐use gut‐on‐a‐chip models with either pre‐seeded Caco‐2 tubules or pre‐seeded adult stem‐cell derived colon organoid tubules. The previously developed workflow with 3 measurement cycles per modulator was followed for the Caco‐2 model. For the colon organoid model, the experiment was limited to a single measurement cycle per modulator, as prior experience indicated that these cells are highly sensitive to experimental manipulations such as medium changes as well as to variations in temperature and flow rate, increasing the risk of cell detachment. When plotting the sensor's fluorescence intensity, we observed the same pattern of oxygen consumption variation in both ready‐to‐use models (Figure 4A,C) as in the Caco‐2‐based model used to develop the Mito Stress test workflow. From these data, changes in maximal OCR in response to the different modulators could be plotted (Figure 4B,D). Since initial seeding densities as well as cellular composition and proliferation rates differed between the two models, absolute comparisons of OCR variation were not meaningful. Changes within each model could nevertheless be compared, as these were calculated relative to the respective baseline respiration of each model. Most notably, a difference in the relative response to FCCP was observed: the Caco‐2‐based model exhibited a 53% increase in OCR upon FCCP addition compared to baseline respiration, whereas the colon organoid model showed a 118% increase, corresponding to a 64 percentage point difference in the relative response between both ready‐to‐use gut‐on‐a‐chip models (Figure 4E).

**FIGURE 4:**
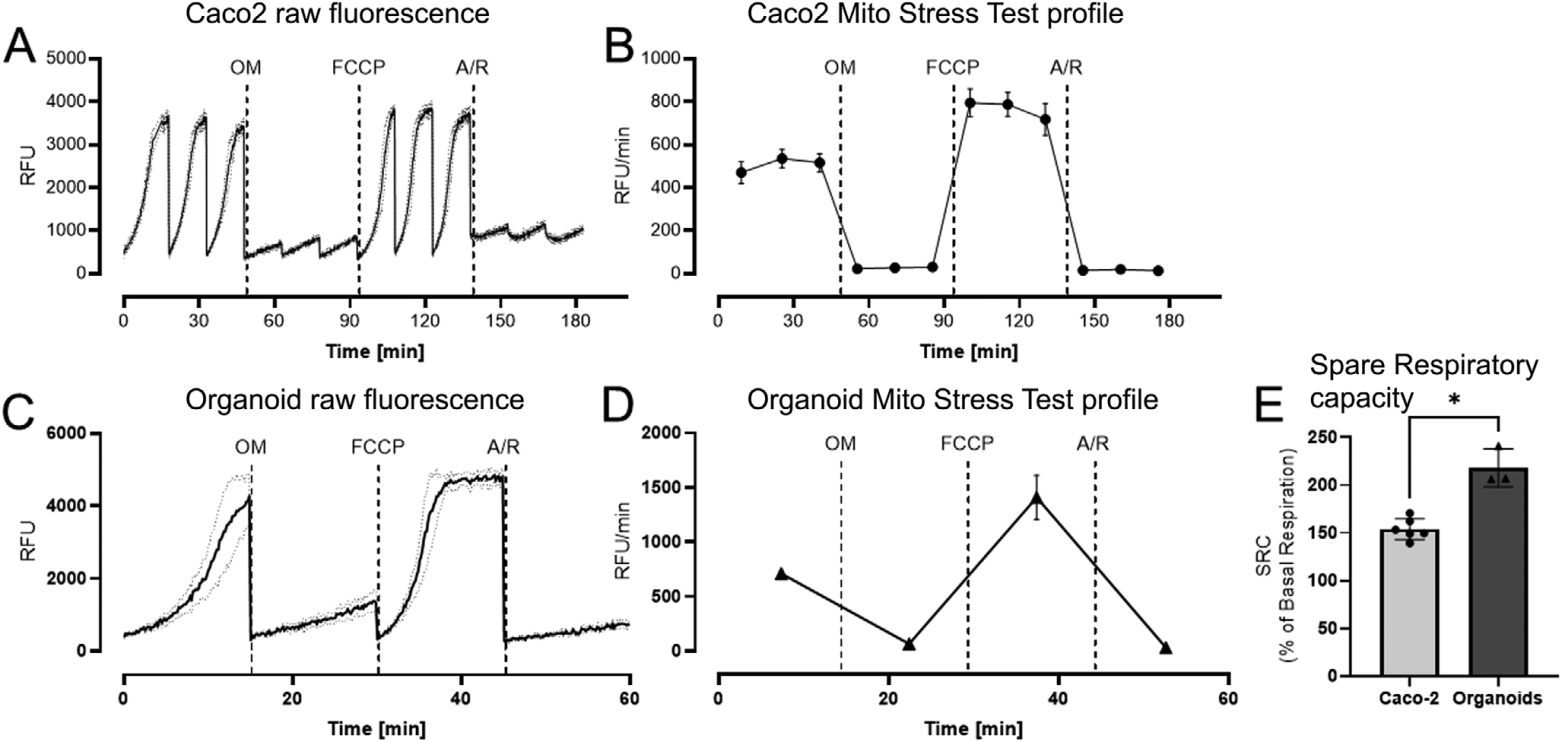
Mitochondrial respiration modulation in ready‐to‐use gut‐on‐a‐chip models. (A) Caco‐2 model. Raw fluorescence intensity expressed as relative fluorescence units (RFU) over time across 3 measurement cycles per modulator (OM; oligomycin, FCCP; carbonyl cyanide p‐trifluoro‐methoxyphenyl hydrazone, A/R; antimycin A/rotenone). Mean (full lines) ± SD (dotted lines) of N = 7, with N = number of chip units. (B) Caco‐2 model. Mito Stress test profile obtained by plotting the maximal OCR of each measurement cycle. Mean (dots) ± SD (error bars) of N = 7 for all measurement cycles except the 3 cycles following FCCP injection, where N = 6, with N = number of chip units. (C) Adult stem‐cell derived colon organoid model. Raw fluorescence intensity expressed as RFU over time across 1 measurement cycle per modulator. Mean (full lines) ± SD (dotted lines) of N = 5, with N = number of chip units. (D). Adult stem‐cell derived colon organoid model. Mito Stress test profile obtained by plotting the maximal OCR of each measurement cycle. Mean (dots) ± SD (error bars) of N = 5 for all measurement cycles except the cycle following FCCP injection, where N = 3, with N = number of chip units. (E) Spare Respiratory Capacity (SRC), calculated as the percentage increase in OCR after FCCP injection compared to baseline for the Caco‐2 and adult stem‐cell derived colon organoid models. Mean ± SD of N = 6 (Caco‐2) or N = 3 (Organoids). *p < 0.05, unpaired t‐test with Welch's correction. Timepoints of compound addition are indicated by dashed lines.

## 4 Discussion

In this study, we validated the use of a liquid‐suspendable metabolic sensor to measure real‐time oxygen consumption in a commercial gut‐on‐a‐chip platform. Most organ‐on‐a‐chip (OoC) devices present analytical challenges such as small medium volumes, low cell numbers and limited internal accessibility, all of which could complicate the use of these sensors that were originally designed for standard well plates [23, 29]. However, initial tests using the sensor in an empty microfluidic device demonstrated detectable fluorescence levels in the medium inlets and outlets with a satisfactory signal‐to‐background ratio. The observed lack of fluorescence increase following glucose oxidase (GOX) addition indicated that oxygen was already depleted to the sensor's maximum detectable signal prior to measurement initiation, which is plausible given the low medium volume and GOX concentration used. Fluorescence measurements from the observation window—the region of biological interest within this OoC system—returned much lower signal‐to‐background values, which is consistent with the substantially smaller volume in this part of the chip. Nevertheless, differences between background and sensor, especially in the presence of GOX, were still quantifiable, with the signal‐to‐background ratio increasing from approximately 2‐fold to 4‐fold upon GOX addition. Although these measurements were performed in a single observation window—and should therefore be considered an initial proof‐of‐concept rather than a replicated sensor performance validation—the observation that the GOX‐induced signal exceeded the manufacturer's recommended 3‐fold signal‐to‐background threshold supports the technical feasibility of detecting oxygen depletion within the observation window. Further support comes from the biological measurements themselves. The reproducible and expected responses to oligomycin, FCCP and antimycin A/rotenone in Figures 3 and 4, all acquired in the observation window, confirm that the sensor detects oxygen changes reliably in this region, beyond the single measurement in Figure 1F. To maximize the sensor's performance, we measured fluorescence in time‐resolved mode, which reduces non‐specific background by temporally separating the short‐lived background fluorescence from the longer‐lived sensor emission, thereby enhancing detection sensitivity. Standard prompt fluorescence intensity measurements can also be performed with this sensor, offering flexibility to use a wide range of commonly available monochromator‐ or filter‐based plate readers. Time‐resolved detection on the other hand, requires more advanced instrumentation in terms of light sources, detectors and timing electronics in order to measure fluorescence decay over time [30, 31]. The need for time‐resolved measurements should be evaluated based on the specific characteristics of the OoC platform.

To evaluate the sensor in an OoC device with intestinal cells inside, a workflow was developed in which Caco‐2 tubules were sequentially exposed to four different respiration modulators. Plotting the maximal oxygen consumption rate throughout the workflow yielded a typical Mito Stress test profile showing the mitochondrial flux parameters (basal, maximal, ATP‐linked and non‐mitochondrial respiration, proton leak and spare respiratory capacity) in a similar pattern to that of a Seahorse Mito Stress test analysis [13]. This experimental workflow was also performed using two commercial gut‐on‐a‐chip models; one containing the human Caco‐2 intestinal epithelial cell line and one containing human colon‐derived organoids, which are composed of a mixture of epithelial cell types derived from intestinal stem cells [26, 32]. Caco‐2 cells are frequently used as in vitro epithelial barrier model systems, although they only represent one intestinal epithelial cell type and therefore do not reflect in vivo physiology [33, 34, 35, 36]. Intestinal organoids, on the other hand, recapitulate the cellular diversity within the epithelium very well, making them a more physiologically relevant option [37]. For both models, the metabolic sensor detected changes in aerobic respiration caused by the mitochondrial modulators. Interestingly, differences in responses between both models could also be identified, as shown by the significantly greater relative respiratory response to FCCP in the organoid model compared to the Caco‐2 model. However, given the different experimental workflows, limited number of measurements for the organoid model and absence of normalization to cell content, this difference should be interpreted as a model‐dependent relative response rather than evidence of intrinsically higher mitochondrial respiratory capacity within the organoid model. Together, these results demonstrate the suitability of this metabolic sensor for established mitochondrial stress testing in OoC devices.

Applicability of the sensor also depends on the microfluidic platform itself. The OrganoPlate used in these experiments has a standard 384‐well plate format and contains a microscopy‐grade glass bottom, making it ideal for both real‐time imaging and measuring. In general, glass‐based microfluidic devices have several benefits that make them highly suitable for oxygen measurements using medium‐soluble compounds. Besides being optically transparent, they also have a very low gas diffusivity, display less non‐specific small molecule absorption and have a lower fluorescence background compared to other frequently used OoC materials such as polymers [38, 39, 40, 41, 42].

A key consideration during these experiments was that measuring required a temporary interruption of microfluidic flow, thereby halting local oxygen replenishment. This constitutes a significant cellular stressor, especially during longer experiments without intermittent re‐introduction of flow, which could affect results. Indeed, our data showed that during baseline respiration, the sensor's fluorescence signal plateaued within minutes. Conversely, measuring without flow interruption would require intermittent medium sampling, causing unwanted re‐oxygenation. The use of a mineral oil overlay to minimize oxygen exchange during sampling could help mitigate this effect [24]. The sensor is cell‐impermeable and inert, and the cells kept their expected respiratory responses throughout the assay, indicating that the sensor did not disturb cellular function during the short measurement window. A dedicated study with paired viability, TEER or morphology assessment before and after sensor exposure would be needed to confirm this for repeated or long‐term measurements. Another important point to emphasize is that, although the sensor's suspendability offers several advantages, it limits spatial localization of oxygen consumption. If additional cell types were to be included, either embedded within the ECM or seeded inside the microfluidic channels, it would not be possible to determine from the collective fluorescence signal within the observation window where oxygen depletion occurred, or whether respiratory activity differed by cell type. Spatial localization of oxygen consumption could be achieved through time‐resolved fluorescence microscopy [43].

In addition to the MitoXpress Xtra sensor, which provides information on aerobic metabolism by responding to changes in oxygen concentration, a similar water‐suspendable, pH‐sensitive, fluorescent pH Xtra sensor exists, which responds to changes in H^+^ concentration, thereby providing information on glycolytic metabolism [25, 44]. Combining both sensors in a dual‐parameter assay, measuring both oxygen consumption and extracellular acidification, would enable simultaneous monitoring of the two main ATP‐generating processes [45]. This could, for example, allow identification of inflammatory (glucose‐driven) or tolerant (oxidative phosphorylation–dependent) metabolic rewiring of immune cells [46]. Another area of OoC research where these sensors could be applied is co‐culture with microbiota, an important domain within intestinal studies that requires precise determination and control of oxygen levels. Oxygen concentrations vary along the gastrointestinal tract both longitudinally as well as radially, which affects bacterial composition [47, 48]. Controlling oxygen concentrations in gut‐on‐a‐chip devices would therefore enable more precise replication of physiological oxygen gradients, support co‐culture with oxygen‐sensitive bacteria and facilitate investigation of host–microbiome interactions. This will ultimately advance the development of microbiome‐related therapeutics, probiotics and nutraceuticals, as well as the evaluation of how changes in intestinal oxygen concentration affect microbial communities [42, 49, 50, 51, 52].

In conclusion, we have demonstrated that a metabolic sensor—soluble in culture medium and thus broadly compatible with diverse OoC platforms—can be effectively combined with the optical properties of a glass‐based microfluidic device to enable non‐invasive, real‐time monitoring of cellular respiration in microliter‐scale volumes. This approach holds significant potential to advance metabolic analysis in microphysiological model systems.

## Author Contributions

**Sarah Devriese**: conceptualization, investigation, writing – original draft, methodology, validation, visualization, writing – review and editing, formal analysis, data curation, project administration. **Flavio Bonanini**: methodology, validation, visualization, writing – review and editing, formal analysis, data curation. **Sara Shafiei**: methodology. **Dorota Kurek**: conceptualization, funding acquisition, methodology, validation, supervision, data curation, project administration. **Coen Govers**: conceptualization, investigation, funding acquisition, writing – review and editing, methodology, project administration, supervision. **Hans Bouwmeester**: conceptualization, investigation, funding acquisition, writing – review and editing, methodology, project administration, supervision. **Remko van Vught**: supervision. **Jaap Keijer**: writing – review and editing, supervision, project administration, conceptualization.

## Funding

This research was supported by the Ministry of Economic Affairs and Climate Policy of the Netherlands via a Public Private Partnership grant from the Top consortium for Knowledge and Life Science & Health, project LSHM21011 “INIMINI‐health: immune‐ and microbiota‐competent intestine‐on‐a‐chip to study health promoting nutrition and drugs”. The following companies contributed to the funding of this project: Agilent Technologies (Cork, Ireland), BioActor B.V. / Solabia Nutrition (Maastricht, The Netherlands), Cargill R&D Centre Europe B.V. (Vilvoorde, Belgium), Mimetas B.V. (Leiden, The Netherlands), Galapagos NV (Mechelen, Belgium) and Winclove Probiotics B.V. (Amsterdam, The Netherlands).

## Conflicts of Interest

FB, DK, SS, and RvV are employees of Mimetas B.V., which commercializes the OrganoPlate and OrganoReady products. For the remaining authors no conflicts of interest were declared.

## Data Availability

The data that support the findings of this study are openly available in zenodo at https://doi.org/10.5281/zenodo.17750978, reference number 17750978.
